# SFRT alleviates symptoms associated with bulky advanced cancer: a retrospective single-center cohort analysis

**DOI:** 10.3389/fonc.2026.1817351

**Published:** 2026-08-24

**Authors:** Haiqin Zhang, Jian Zhang, Chen Su, Yanming Wang, Chuanyong Liu, Yan Dou, Jie Liu, Weichong Zhao, Sen Liu, Changsheng Cong, Jin Yu, Meili Sun

**Affiliations:** Department of Oncology, Central Hospital Affiliated to Shandong First Medical University, Jinan, Shandong, China

**Keywords:** bulky tumor, immunotherapy, lattice radiotherapy, spatially fractionated radiation therapy, stereotactic central/core ablative radiation therapy

## Abstract

**Objective/background:**

To evaluate the impact of Spatially Fractionated Radiation Therapy (SFRT) on symptomatic relief and survival outcomes in patients with large-volume tumors.

**Methods:**

A total of 60 cancer patients who received SFRT between February 2023 and May 2025 were analyzed in this retrospective study. Collected data comprised demographic characteristics, as well as follow-up information on symptomatic response, best overall response, survival duration, and treatment-related adverse events.

**Results:**

By the end of the median follow-up duration of 15.8 months (3.8–30.8 months), 91.7% (55/60) of patients showed symptomatic relief. Follow-up imaging was available for 51 patients, showing tumor regression in 45 cases (6 showed increased volume after treatment). According to RECIST 1.1 criteria, 17 patients achieved Partial response (PR), 32 had Stable disease (SD), and 2 experienced Progressive disease (PD). The Overall response rate (ORR) was 33.3%, and the Disease control rate (DCR) was 96.1%. The median Overall survival (OS) was 7.6 months (95% CI: 4.3-10.9). Univariate analysis identified that a Biologically effective dose at the Vertex (BED-Vertex) ≥ 88 Gy was significantly associated with treatment response (P = 0.020). All adverse events were mild to moderate in severity, no grade 3 or higher toxicities were observed.

**Conclusion:**

SFRT appeared to be effective with limited toxicities for bulky tumors. BED-Vertex is a significant predictor of response in univariate analysis, suggesting that a strategy of safe BED escalation could enhance palliative efficacy.

## Introduction

Recent advances in immunotherapy, cell therapy, targeted drugs, precision radiotherapy, and multidisciplinary care have markedly improved cancer treatment outcomes and prolonged patient survival (1–7). Nevertheless, bulky tumors remain a major therapeutic challenge in modern oncology (7, 8). The hostile microenvironment within these tumors—characterized by hypoxia, abnormal vasculature, and metabolic stress—confers inherent treatment resistance, hindering the efficacy of conventional therapies including conventional fractionated radiation, chemotherapy, targeted agents, and immunotherapy (6, 9–11).

Spatially Fractionated Radiation Therapy (SFRT) is an emerging technique that shows great potential for bulky tumors (12–17). It can deliver highly focused, ablative doses to tumor subregions with minimal organ exposure. This intentional dose heterogeneity generates diverse tumor antigens to boost immune activation, while sparing lymphoid-rich margins that may harbor anti-tumor immune cells. However, the literature on this strategy is sparse (18–24). We therefore conducted a retrospective review of two-year data from our center, aimed at bridging this clinical evidence gap.

## Methods and analysis

### Patients

This retrospective, single-center study was conducted to evaluate the efficacy and safety of SFRT for bulky tumors. It was approved by our Institutional Ethics Committee (Approval No. 20251223002) and conducted in accordance with the Declaration of Helsinki.

From February 2023 to May 2025, 60 patients with bulky tumors (25)—defined as parenchymal lesions ≥5 cm in maximal diameter or metastatic lymph nodes ≥2 cm in short-axis diameter—who underwent SFRT at the Department of Oncology of Central hospital affiliated to Shandong First Medical University were enrolled. All enrolled patients were either pretreated advanced cases with no further standard therapeutic options, or patients with highly symptomatic local disease.

### Treatment and follow-up

The radiation oncologist tailors the radiotherapy regimen—encompassing dosage, technique, and equipment—based on key clinical determinants such as tumor location and the radiation tolerance of adjacent organs. Two principal techniques are applied according to tumor geometry. For voluminous, regularly shaped tumors, the Stereotactic Central/Core Ablative Radiation Therapy (SCART) technique is indicated, characterized by a Vertex target volume generated by applying a uniform 1.5 - 2.0 cm isotropic margin. For irregular or elongated morphologies, the Lattice Radiotherapy (LRT) technique is used. Vertices placement integrated both geometric and biological considerations. Spherical vertices with a diameter of 0.5-1.5 cm were created within the Gross Tumor Volume (GTV), ensuring an inter-vertex center distance of ≥ 3 cm and a safety margin of ≥ 2 cm from adjacent Organs at Risks (OARs) (26–28).

### Assessment of SFRT

Treatment response is evaluated through multi-faceted endpoints: pain relief is defined as a reduction in the Numerical Rating Scale (NRS) score with corresponding downgrading in severity, and hemorrhagic control is confirmed by the cessation of grossly visible bleeding. Alleviation of other symptoms is assessed through a comprehensive analysis of patient-reported outcomes, correlative imaging, and laboratory data. Antitumor efficacy, specifically regarding tumor burden, is objectively evaluated by diagnostic imaging (CT or MRI) in accordance with Response Evaluation Criteria In Solid Tumors (version 1.1) (RECIST 1.1) criteria. Treatment efficacy is evaluated through key oncology endpoints: the Objective response rate (ORR), defined as the proportion of patients with a best overall response of Complete response (CR) or Partial response (PR); the Disease control rate (DCR), which encompasses patients achieving CR, PR, and SD; and the Overall survival (OS), measuring the time from SFRT treatment initiation until death from any cause. Adverse events were classified according to the Common Terminology Criteria for Adverse Events version 5.0 (CTCAE 5.0).

### Statistical analysis

Statistical analyses were performed using R software (version 4.4.2; R Foundation for Statistical Computing, Vienna, Austria) and R Studio (version 2024.12.1 + 563; Posit Software, PBC, Boston, MA). Specifically, the analyses were conducted following the guidelines of the R Development Core Team (2024). Categorical variables were expressed as counts and percentages, and comparisons were made using Pearson’s Chi-square test or Fisher’s exact test, as appropriate. Survival curves were drawn using the Kaplan-Meier method. Patient demographics, tumor types, and radiotherapy parameters were included in univariate logistic regression models to evaluate variables associated with the best overall response. The biologically effective dose (BED) for the vertex was calculated using the linear-quadratic model (denoted as BED-Vertex), while the BED for the entire tumor was calculated using the Niemierko model (denoted as BED-GTV) with an a-value of −10 and an α/β ratio of 10 Gy. The cutoff values of age, BED for analysis were set to the median values. P < 0.05 was considered being indicative of statistical significance.

## Results

### Patient characteristics

The characteristics of the 60 patients included in the analysis are shown in **Table 1**. The study population included 38 males and 22 females, with a median age of 66 years (range: 26–88 years); 30 patients (50%) had an ECOG PS of 0 to 1. The cohort was composed of 14 distinct primary tumor types, with lung cancer (17 cases, 28.3%), colorectal cancer (8 cases, 13.3%), and soft tissue sarcoma (7 cases, 11.7%) being the most frequent.

**Table 1:** Patient characteristics (n=60).

Clinical features	Value
Median Age (Minimum - Maximum)	66 years (26-88)
Sex n(%)	
Male	38 (63.3)
Female	22 (36.7)
PS (ECOG) n (%)	
0-1	30 (50.0)
≥2	30 (50.0)
Primary disease n (%)	
Lung Cancer	17 (28.3)
Colorectal Cancer	8 (13.3)
Soft Tissue Sarcoma	7 (11.7)
Liver Cancer	5 (8.3)
Pancreatic Cancer	5 (8.3)
Cholangiocarcinoma	3 (5.0)
Urothelial Carcinoma	3 (5.0)
Gastric Cancer	3 (5.0)
Thyroid Cancer	2 (3.3)
Ovarian Cancer	2 (3.3)
Renal Cancer	2 (3.3)
Prostatic Cancer	1 (1.7)
Esophageal Cancer	1 (1.7)
Head and Neck Squamous Cell Carcinoma	1 (1.7)
Lesion location n (%)	
Liver	20 (33.3)
Soft Tissue	14 (23.3)
Lung	11 (18.3)
Lymph Node	4 (6.7)
Pancreas	4 (6.7)
Adrenal Gland	3 (5.0)
Bladder	1 (1.7)
Thyroid Gland	1 (1.7)
Ovary	1 (1.7)
Portal Vein Tumor Thrombus (PVTT)	1 (1.7)
Clinical stages n (%)	
I	3 (5.0)
II	1 (1.7)
III	3 (5.0)
IV	53 (88.3)
Classical symptoms (%)	
Pain	41 (68.3)
Dyspnea	13 (21.7)
Haemorrhage	2 (3.3)
Tenesmus	2 (3.3)
Bowel Obstruction	1 (1.7)
Portal Hypertension	1 (1.7)
Number of treatment lines n (%)	
1	23 (38.3)
2	16 (26.7)
3	7 (11.7)
4	6 (10.0)
5	3 (5.0)
6	3 (5.0)
9	2 (3.3)

The most common irradiation sites were the liver (20 cases, 33.3%), soft tissue (14 cases, 23.3%), and lung (11 cases, 18.3%), followed by lymph nodes and pancreas (4 cases each, 6.7%), adrenal gland (3 cases, 5.0%), and bladder, thyroid, ovary, and portal vein tumor thrombus (1 case each, 1.7%). A high proportion of patients (53 cases, 88.3%) had American Joint Committee on Cancer (AJCC) stage IV disease. Regarding treatment history, 23 (38.3%) were treatment-naive and 37 (61.7%) had received ≥first-line therapy. All enrolled patients were either pretreated advanced cases with no further standard therapeutic options, or patients with highly symptomatic local disease. All anti-tumor treatment regimens were approved by the Multidisciplinary team (MDT).

Radiotherapy was delivered with palliative intent for a range of clinical indications, including pain (41 cases, 68.3%), dyspnea (13 cases, 21.7%), tenesmus and hemorrhage (2 cases each, 3.3%), as well as intestinal obstruction and portal hypertension (1 case each, 1.7%).

The median pretreatment tumor diameter was 83.55 mm (range: 36.43–224.50 mm), and median tumor volume was 288.20 cm³ (range: 33.04–2,209.73 cm³).

18 patients were treated with LRT and 42 with SCART technique. A summary of all fractionation regimens was shown in **Supplementary Table 1**. The median BED-Vetex was 88 Gy (range, 40–195 Gy). All patients underwent SFRT with daily Cone beam computed tomography (CBCT) guidance.

### Effectiveness

**Figure 1:**
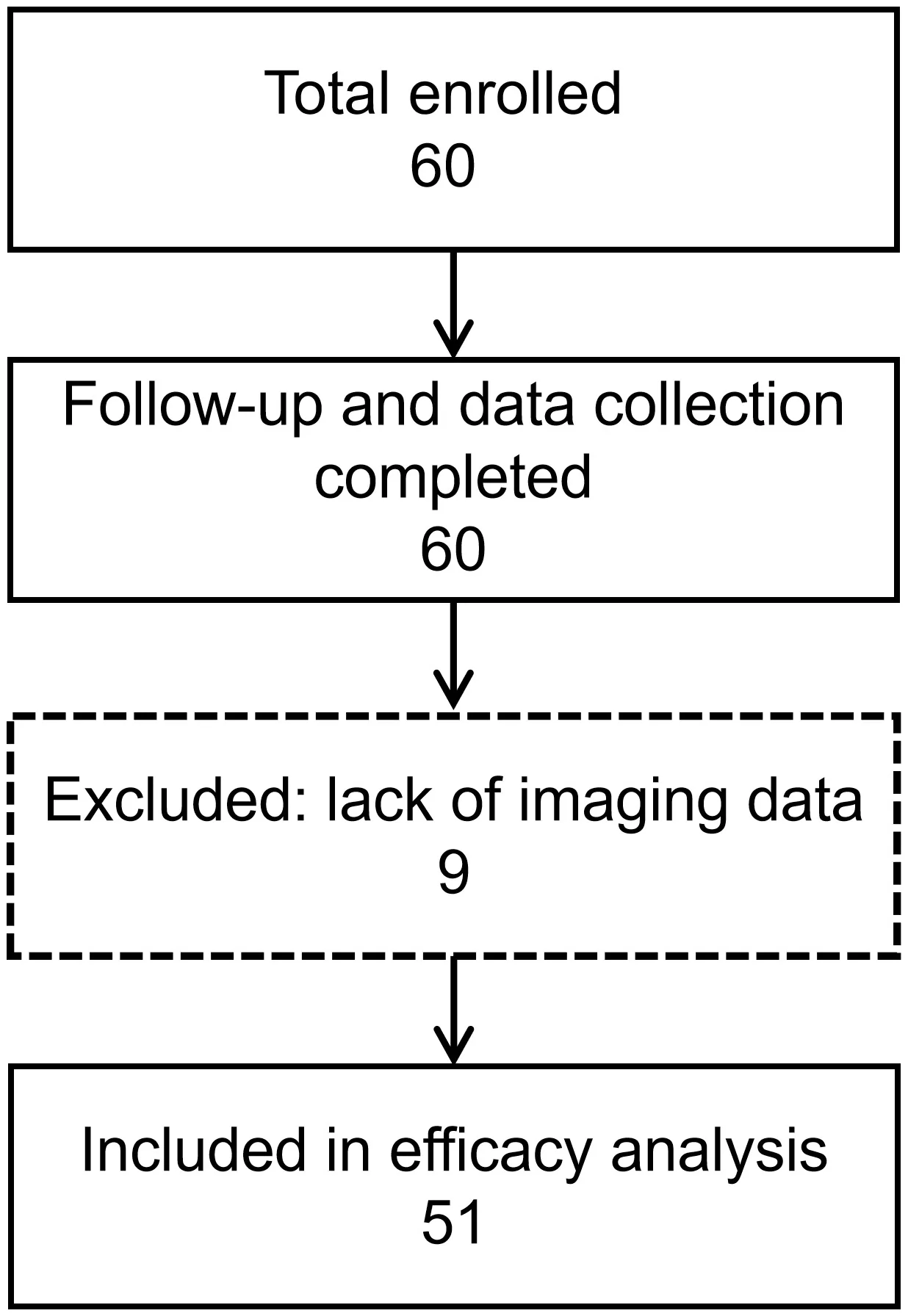
CONSORT-style flow diagram of patient selection and inclusion for the efficacy analysis. A total of 60 patients were initially enrolled and completed follow-up data collection. Nine patients were excluded from the efficacy analysis due to insufficient imaging data for response assessment.

**Figure 2:**
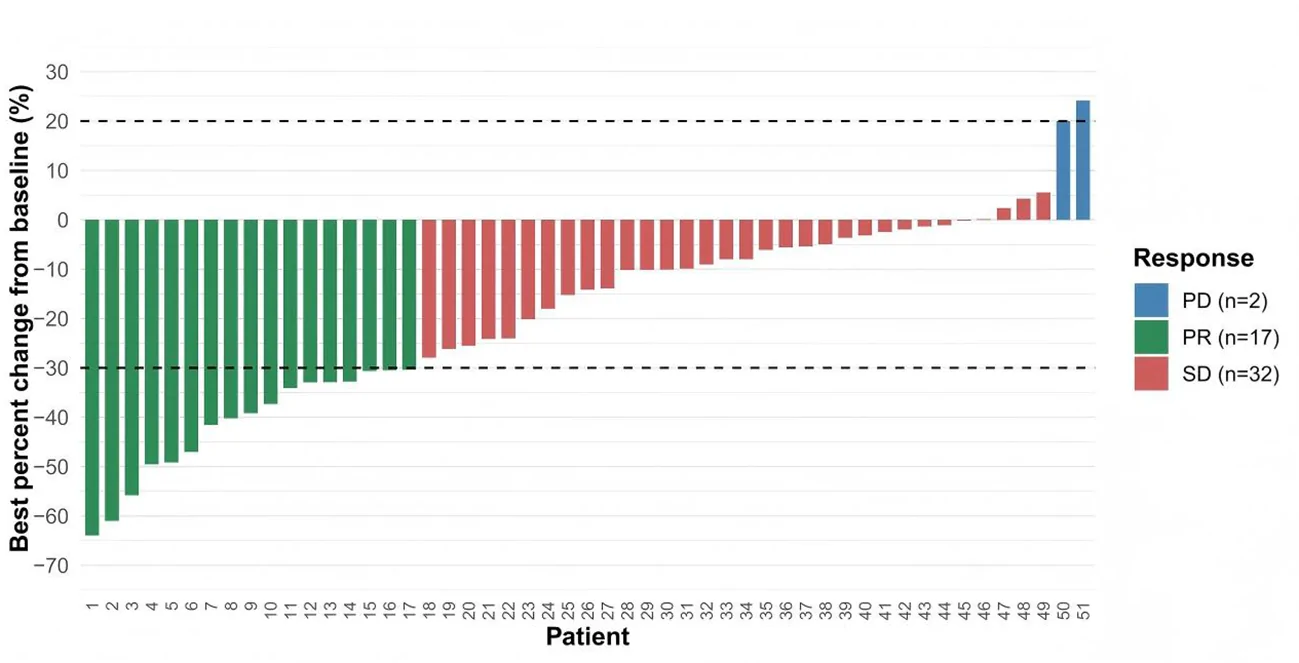
Waterfall plot for target lesion tumor size. 51 patients were included in the efficacy analyses. Tumor regression was observed in 45 cases, with the remaining 6 showing enlargement. The best overall response per RECIST 1.1 was a confirmed partial response (PR) in 17 patients, stable disease (SD) in 32, and progressive disease (PD) in 2.

**Figure 3:**
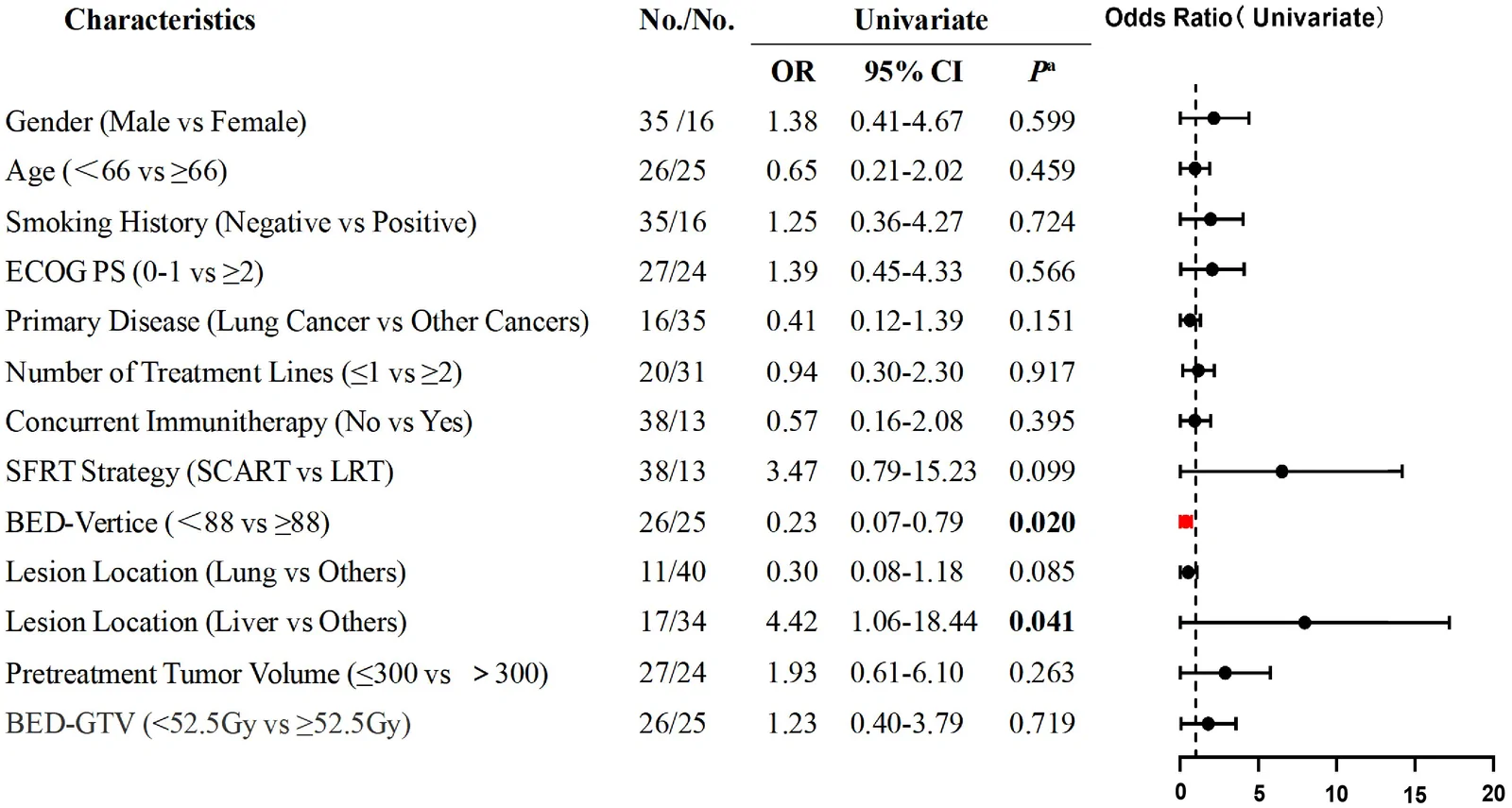
Univariate analysis of factors associated with treatment response. ^a^P-values from univariate logistic regression models for ordinal outcome PR/SD/PD. Abbreviations and Note: PR: Partial response; SD: Stable disease; PD: Progressive disease; 95% CI: 95% confidence interval; OR: Odds ratio.

Symptomatic relief was evaluated 2 to 4 weeks after radiotherapy. As is shown in **Table 2**, the overall relief rate was 91.7% (55/60), with specific rates as follows: pain 92.7% (38/41), dyspnea 100% (13/13), hemorrhage 100% (2/2), tenesmus 100% (2/2), bowel obstruction 0% (0/1) and portal hypertension 0% (0/1). Baseline and post-treatment severity of classical symptoms was shown in **Supplementary Table 2**. Tumor regression and associated symptom improvement typically occurred within an average of 1 to 3 weeks. As is shown in **Figure 1**, follow-up imaging was available for 51 patients. The first reassessment generally occurred within 3 months post-treatment. Response evaluation was standardized using RECIST 1.1 criteria to calculate ORR and DCR. Tumor regression was observed in 45 cases (88.2%), with the remaining 6 (11.8%) showing enlargement. **Figure 2** displays a waterfall plot illustrating the change in target lesion diameters from baseline. As summarized in **Table 3**, the best overall response was a confirmed PR in 17 patients (33.3%), Stable disease (SD) in 32 (62.7%), and Progressive disease (PD) in 2 (3.9%), corresponding to an ORR of 33.3% and a DCR of 96.1%. The results of the univariate ordinal logistic regression analysis identifying factors associated with treatment response are shown in **Figure 3**. A BED-Vertex ≥ 88 Gy was identified as a significant predictor of treatment response in the univariate analysis (P = 0.020).

**Table 2:** Symptomatic relief after SFRT.

Classical symptoms	Symptomatic relief rate (%)
Pain	38/41 (92.7)
Dyspnea	13/13 (100.0)
Haemorrhage	2/2 (100.0)
Tenesmus	2/2 (100.0)
Bowel Obstruction	0/1 (0.0)
Portal Hypertension	0/1 (0.0)
Amount To	55/60 (91.7)

**Table 3:** Distribution of baseline characteristics by best overall response based on RECIST 1.1 criteria.

Clinical features	Overall	PR	SD	PD
	(n=51)	(n=17)	(n=32)	(n=2)
Gender (%)				
Male	35	13 (37.1)	20 (57.1)	2 (5.7)
Female	16	4 (25.0)	12 (75.0)	0 (0.0)
Age (%)				
<66	26	8 (30.8)	16 (61.5)	2 (7.7)
≥66	25	9 (36.0)	16 (64.0)	0 (0.0)
Smoking history (%)				
Positive	35	12 (34.3)	22 (62.9)	1 (2.9)
Negative	16	5 (31.3)	10 (62.5)	1 (6.3)
ECOG PS (%)				
0-1	27	10 (37.0)	16 (59.3)	1 (3.7)
≥2	24	7 (29.2)	16 (66.7)	1 (4.2)
Primary disease (%)				
Lung Cancer	16	8 (50.0)	7 (43.8)	1 (6.3)
Other Cancers	35	9 (25.7)	25 (71.4)	1 (2.9)
Number of treatment lines (%)				
≤1	20	7 (35.0)	12 (60.0)	1 (5.0)
≥2	31	10 (32.3)	20 (64.5)	1 (3.2)
Concurrent immunotherapy (%)				
No	38	11 (28.9)	26 (68.4)	1 (2.6)
Yes	13	6 (46.2)	6 (46.2)	1 (7.7)
SFRT strategy (%)				
SCART	38	15 (39.5)	22 (58.9)	1 (2.6)
LRT	13	2 (15.4)	10 (76.9)	1 (7.7)
BED-vertice (%)				
<88	26	5 (19.2)	19 (73.1)	2 (7.7)
≥88	25	12 (48.0)	13 (52.0)	0 (0.0)
Lesion location (%)				
Lung	11	6 (54.5)	5 (45.5)	0 (0.0)
Others	40	11 (27.5)	27 (67.5)	2 (5.0)
Lesion location (%)				
Liver	17	3 (17.6)	12 (70.6)	2 (11.8)
Others	34	14 (41.2)	20 (58.8)	0 (0.0)
Pretreatment tumor volume (%)				
≤300	27	11 (40.7)	15 (55.6)	1 (3.7)
>300	24	6 (25.0)	17 (70.8)	1 (4.2)
BED-GTV (%)				
<52.5Gy	26	8 (30.8)	17 (65.4)	1 (3.8)
≥52.5Gy	25	9 (36.0)	15 (60.0)	1 (4.0)

**Figure 4:**
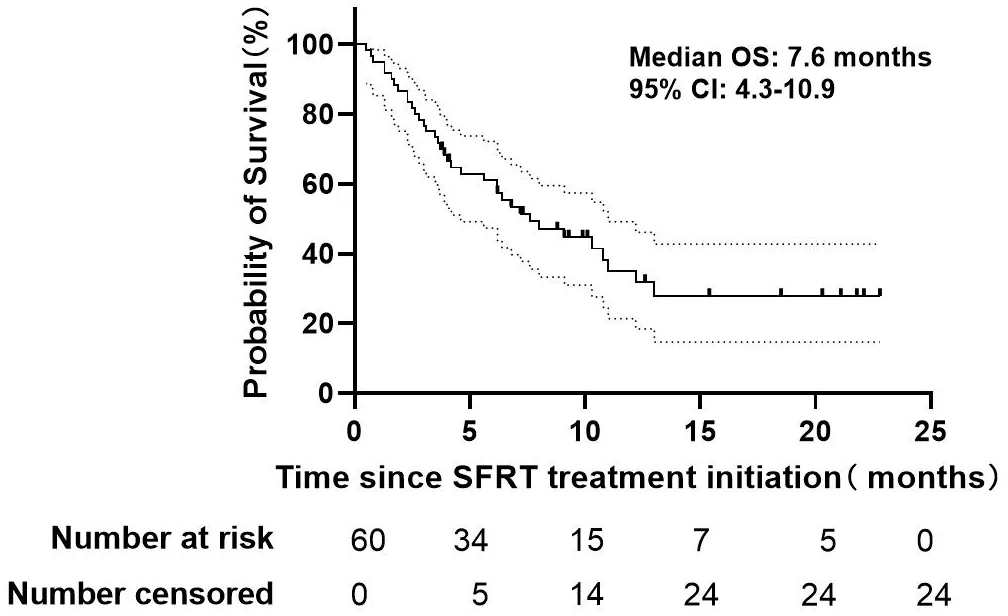
Kaplan–Meier survival curves depicting the duration of overall survival in months. With a median follow-up of 15.8 months, 36 deaths were recorded among the 60-patient cohort. OS is defined as time from SFRT treatment initiation until death from any cause. CI for median was computed using the Brookmeyer and Crowley method vertical marks indicate censored data to the time of an event. CI: confidence inteval; OS, Overall survival.

At the data cut-off (September 17, 2025), with a median follow-up of 15.8 months, 36 deaths were recorded among the 60-patient cohort. The median OS was 7.6 months (95% CI: 4.3-10.9) (**Figure 4**).

### Safety

None of the patients developed grade 3 or higher toxicities. As is shown in **Table 4**, the toxicities among the 60 patients included 3 (5%) patients with grade 1 radiation pneumonitis, 3 (5%) patients with grade 1 hepatotoxicity, 2 (3.3%) patients with grade 2 dermatitis, 1 (1.7%) patient with grade 1 gastrointestinal toxicity, and 1 (1.7%) patient with grade 2 infection.

**Table 4:** Summary of adverse events graded by CTCAE v5.0.

Adverse events	Incidence (%)
Grade 2 Dermatitis	2/60 (3.3)
Grade 2 Infection	1/60(1.7)
Grade 1 Radiation Pneumonitis	3/60(5.0)
Grade 1 Hepatotoxicity	3/60(5.0)
Grade 1 Gastrointestinal Toxicity	1/60(1.7)

## Discussion

This single-center retrospective study demonstrates that SFRT may represent an effective palliative therapeutic approach with an acceptable toxicity profile for managing bulky tumors.

As previously indicated, bulky tumors are characterized not only by their substantial volume but also by a constellation of challenging features, including high aggressiveness, rapid growth, hypoxia, extensive prior treatment history, resistance to radiotherapy, chemotherapy, and immunotherapy, close proximity to critical normal structures, poor patient performance status, and refractory symptoms (7–10, 29–32). These factors collectively contribute to the considerable difficulty in their clinical management. The patient cohort in this study exhibited significant heterogeneity, with the majority having undergone multiple prior interventions, including surgery, radiotherapy, and systemic therapies. The tumor spectrum was notably diverse, encompassing 10 primary or metastatic sites originating from 14 distinct solid tumor types, including lung cancer, colorectal cancer, soft tissue sarcoma, among others. The liver, soft tissue, and lungs constituted the most frequently irradiated sites. This diversity preliminarily illustrates the applicability of SFRT across various solid tumors and suggests its potential for broader clinical generalization.

In our study, we included two SFRT techniques: LRT and SCART. While LRT creates a three-dimensional array of high-dose vertices, SCART delivers highly focused, ablative doses specifically to the tumor core while intentionally sparing the periphery (20, 33–35). Despite these geometric differences, both techniques fundamentally share the same core dosimetric intent: creating intentional intratumoral dose heterogeneity characterized by high-dose “peaks” and low-dose “valleys” (36, 37). This non-uniform dose distribution not only minimizes exposure to surrounding organs but also generates diverse tumor antigens to boost immune activation through non-conventional radiobiological mechanisms, such as the bystander effect and vascular damage (38–40). Furthermore, this spatial fractionation spares lymphoid-rich margins that may harbor anti-tumor immune cells.

Our findings underscore the considerable therapeutic potential and favorable safety profile of SFRT. In the context of palliation, rapid symptomatic relief—including alleviation of pain, dyspnea, and bleeding—was achieved in 91.7% of cases, significantly improving patients’ quality of life and potentially enhancing their overall performance status. Notably, our study demonstrated rapid tumor regression and associated symptom improvement, suggesting a potentially faster onset of action compared to conventional palliative radiotherapy or chemotherapy. An ORR of 33.3% and a DCR of 96.1% were observed. Consistent with previous literature, no Grade 3 or higher adverse events were reported, a finding attributable to SFRT’s unique dose distribution paradigm, which selectively targets specific intra-tumoral regions (e.g., “central zones”) while effectively sparing surrounding normal tissues and organs from high-dose exposure.

In our study, patients with immunotherapy got a numerically higher PR compared to those without immunotherapy. The synergistic potential between radiotherapy and immunotherapy has garnered significant attention in recent years. The landmark PACIFIC phase 3 trial (41) in patients with stage III Non-Small Cell Lung Cancer (NSCLC) heralded a new era for combining radiotherapy with Immune Checkpoint Inhibitors (ICIs) targeting the Programmed Death-1 (PD-1)/Programmed Death-Ligand 1 (PD-L1) axis. However, numerous subsequent clinical trials combining conventional radiotherapy with immunotherapy have yielded negative results (42–45). Key contributing factors include the inclusion of large volumes such as draining lymph nodes and blood pools within radiation fields, lymphodepletion caused by extended fractionation schedules, and the potential for vascular damage and hypoxia induced by conventional radiotherapy to foster an immunosuppressive microenvironment (46, 47). Recent clinical evidence, ranging from early-stage to metastatic disease, demonstrates that combining Stereotactic Body RadioTtherapy (SBRT) or Stereotactic Ablative Radiotherapy (SABR) (versus conventional radiotherapy) with immunotherapy yields superior outcomes in event-free survival, progression-free survival, overall survival, and out-of-field response rates (48–52). Beyond ablative doses, low-dose radiotherapy was believed to reprogram the tumor immune microenvironment by promoting vascular normalization, recruiting immune effector cells, and enhancing antigen presentation (53–57). This rationale supports a promising combinatorial strategy: pairing high-dose radiotherapy (to generate an *in situ* vaccine) with low-dose radiotherapy (to condition the microenvironment) and immune checkpoint blockade (to sustain the T-cell response), thereby eliciting potent systemic and local anti-tumor immunity (58). Recent studies indicate that combining radiotherapy with ICIs may enable patients to continue systemic therapy even after disease progression, thereby improving survival outcomes, even among those with innate resistance (59).

Notably, SFRT intrinsically embodies this concept through its characteristic “peak-and-valley” dose distribution. Within the same tumor volume, the high-dose “peaks” achieve significant cytoreduction and antigen release, while the intentionally spared low-dose “valleys” preserve critical stromal structures and vasculature. These preserved channels are hypothesized to facilitate the infiltration, survival, and function of key immune cells, such as CD8+ T cells and tissue-resident memory T cells, potentially amplifying a systemic immune response capable of inhibiting untreated distant metastases—the abscopal effect (60, 61). Therefore, SFRT represents a highly promising platform for combination with immunotherapy and targeted agents. Anecdotal clinical reports even suggest its potential to overcome resistance to targeted or immunotherapies, offering a novel therapeutic avenue for heavily pretreated patients (62). In our study, a numerically higher PR rate was observed in patients receiving concurrent immunotherapy (46.15%) compared to those who did not (28.95%), although this difference did not reach statistical significance. This preliminary finding warrants further investigation in larger, prospective studies.

A key finding of our study was the significant association between a higher cumulative BED-Vertex and improved treatment response identified in the univariate analysis. Patients receiving a cumulative BED-Vertex ≥88 Gy demonstrated significantly better outcomes, with PR, SD, and PD rates of 48.0%, 52.0%, and 0.0%, respectively, compared to 19.2%, 73.1%, and 7.7% in those with BED <88 Gy (P = 0.020). This strong dose-response relationship underscores that achieving a higher cumulative dose at vetex is a critical determinant of local tumor control with SFRT, providing valuable preliminary clinical evidence for dose optimization in bulky tumors (63).

Several limitations of this study must be acknowledged. First, its retrospective design inherently introduces selection bias and limits the ability to infer causality. Second, the cohort exhibited significant heterogeneity in primary tumor types, histology, prior treatments, lesion locations, as well as in the delivered BED and fractionation schemes, which complicates direct comparisons and generalizability. Third, a major limitation specific to SFRT is the lack of standardization, which limits reproducibility across institutions. Because this study is derived from heterogeneous clinical data, it inherently lacks the strict dosimetric standardization found in prospective trials. Consequently, detailed parameters such as peak-to-valley dose ratio (PVDR), equivalent uniform dose (EUD), vertex dimensions, valley dose, conformity metrics, and gradient indices were not uniformly recorded or optimized in the original treatment plans, making comprehensive retrospective reconstruction difficult. Furthermore, the assessment of outcomes was constrained by a relatively small sample size and a low number of events, resulting in limited statistical power and wide confidence intervals in certain analyses. From a radiobiological perspective, we applied a uniform α/β ratio of 10 Gy to calculate BED across the entire cohort. While this value serves as a standardized approximation for tumor control consistent with previous SBRT/SFRT studies, it represents a simplification. Applying a fixed α/β ratio may not accurately reflect the distinct radiobiological characteristics of all tumor subtypes included in this mixed cohort, suggesting that the actual biological effective dose might vary by histology. Additionally, suboptimal imaging follow-up for some patients may have led to an underestimation of the best objective response, while inconsistencies in toxicity documentation hindered a comprehensive safety evaluation. The retrospective assessment of subjective endpoints like symptomatic relief is also particularly susceptible to bias. Finally, the variable follow-up may have led to underreporting of late toxicities or recurrences.

Despite these limitations, our analysis provides important preliminary evidence regarding the feasibility and clinical benefits of SFRT for bulky tumors, particularly in patients requiring urgent local control. Future prospective studies are warranted to validate these findings using rigorous protocols that explicitly report and analyze correlations between clinical outcomes and key dosimetric parameters, thereby optimizing the clinical application of SFRT.

## Conclusion

Our findings suggest that SFRT is an effective and well-tolerated palliative therapeutic option for managing bulky tumors. In univariate analysis, a cumulative BED ≥88 Gy was significantly associated with optimal treatment response A low incidence of severe toxicities further supports the safety of this approach. However, variations in its definition and treatment protocols highlight the need for standardized guidelines. Prospective studies are warranted to confirm these findings and to define the optimal role of SFRT in clinical practice.

## Data Availability

The original contributions presented in the study are included in the article/**Supplementary Material**. Further inquiries can be directed to the corresponding author.
