# Supplementary material for: SFRT alleviates symptoms associated with bulky advanced cancer: a retrospective single-center cohort analysis
Source: Front Oncol. 2026 Aug 24;16:1817351. doi: 10.3389/fonc.2026.1817351 (PMC13547061; doi:10.3389/fonc.2026.1817351)
Supplement: Supplementary file 1 [file Table1.docx]

**Supplementary Table 1. A Summary of All Fractionation Regimens**

| No. | Peak dose | Synchronous/Sequential | Valley dose |
| --- | --- | --- | --- |
| 1 | 21Gy×3f | Synchronous | 5Gy×3f |
| 2 | 18Gy×1f | Sequential | 2Gy×25f |
| 3 | 15Gy×1f | Sequential | 2Gy×25f |
| 4-5 | 15Gy×1f | Sequential | 2Gy×20f |
| 6-8 | 15Gy×1f | Sequential | 2Gy×25f |
| 9 | 12Gy×3f | Sequential | 2Gy×20f |
| 10 | 12Gy×3f | Synchronous | 3Gy×3f→3Gy×7f |
| 11-16 | 12Gy×1f | Sequential | 2Gy×20f |
| 17 | 12Gy×1f | Sequential | 3Gy×10f |
| 18 | 12Gy×1f | Sequential | 1.8Gy×28f |
| 19 | 12Gy×1f | Sequential | 1.8Gy×25f |
| 20-21 | 12Gy×1f | Sequential | 2Gy×25f |
| 22 | 10Gy×5f | Sequential | 3Gy×10f |
| 23-26 | 10Gy×3f | Sequential | 3Gy×10f |
| 27-28 | 10Gy×3f | Sequential | 2Gy×20f |
| 29 | 10Gy×2f | Sequential | 3Gy×10f |
| 30-33 | 10Gy×2f | Sequential | 3Gy×10f |
| 34 | 10Gy×2f | Synchronous | 3Gy×2f |
| 35-37 | 10Gy×2f | Sequential | 2Gy×20f |
| 38-41 | 10Gy×1f | Sequential | 2Gy×20f |
| 42-43 | 10Gy×1f | Sequential | 3Gy×10f |
| 44 | 10Gy×1f | Sequential | 1.8Gy×25f |
| 45 | 9Gy×3f | Synchronous | 2Gy×3f→2Gy×17f |
| 46-47 | 9Gy×3f | Sequential | 2Gy×20f |
| 48 | 8Gy×3f | Sequential | 2Gy×25f |
| 49 | 8Gy×3f | Sequential | 3Gy×10f |
| 50 | 7Gy×8f | Synchronous | 2Gy×8f→2Gy×12f |
| 51 | 7Gy×8f | Synchronous | 3Gy×8f |
| 52-55 | 6Gy×10f | Synchronous | 3Gy×10f |
| 56 | 6Gy×10f | Synchronous | 2Gy×10f |
| 57-59 | 6Gy×10f | Synchronous | 3Gy×10f |
| 60 | 5Gy×10f | Synchronous | 2Gy×10f |

**Supplementary Table 2. Baseline and Post-treatment Severity of Classical Symptoms**

| Classical Symptoms | Number | Baseline Severity | Post-treatment Severity | Symptomatic Relief (Y/N) |
| --- | --- | --- | --- | --- |
| Pain (NRS) | 1 | 5 | 2 | Y |
|  | 2 | 6 | 0 | Y |
|  | 3 | 4 | 2 | Y |
|  | 4 | 4 | 2 | Y |
|  | 5 | 5 | 3 | Y |
|  | 6 | 7 | 2 | Y |
|  | 7 | 4 | 2 | Y |
|  | 8 | 9 | 2 | Y |
|  | 9 | 7 | 3 | Y |
|  | 10 | 4 | 2 | Y |
|  | 11 | 7 | 3 | Y |
|  | 12 | 5 | 2 | Y |
|  | 13 | 8 | 2 | Y |
|  | 14 | 4 | 2 | Y |
|  | 15 | 4 | 2 | Y |
|  | 16 | 4 | 1 | Y |
|  | 17 | 5 | 4 | Y |
|  | 18 | 7 | 4 | N |
|  | 19 | 4 | 2 | Y |
|  | 20 | 6 | 1 | Y |
|  | 21 | 4 | 1 | Y |
|  | 22 | 4 | 1 | Y |
|  | 23 | 4 | 3 | Y |
|  | 24 | 5 | 3 | Y |
|  | 25 | 4 | 3 | Y |
|  | 26 | 4 | 2 | Y |
|  | 27 | 6 | 4 | N |
|  | 28 | 7 | 1 | Y |
|  | 29 | 8 | 2 | Y |
|  | 30 | 4 | 2 | Y |
|  | 31 | 8 | 3 | Y |
|  | 32 | 4 | 2 | Y |
|  | 33 | 4 | 2 | Y |
|  | 34 | 5 | 3 | Y |
|  | 35 | 9 | 6 | Y |
|  | 36 | 9 | 2 | Y |
|  | 37 | 4 | 0 | Y |
|  | 38 | 6 | 3 | Y |
|  | 39 | 7 | 2 | Y |
|  | 40 | 4 | 2 | Y |
|  | 41 | 6 | 7 | N |
| Dyspnea (CTCAE) | 42 | 3 | 1 | Y |
|  | 43 | 3 | 1 | Y |
|  | 44 | 4 | 2 | Y |
|  | 45 | 2 | 0 | Y |
|  | 46 | 3 | 1 | Y |
|  | 47 | 3 | 1 | Y |
|  | 48 | 2 | 1 | Y |
|  | 49 | 3 | 2 | Y |
|  | 50 | 3 | 1 | Y |
|  | 51 | 2 | 1 | Y |
|  | 52 | 2 | 1 | Y |
|  | 53 | 3 | 0 | Y |
|  | 54 | 1 | 0 | Y |
| Haemorrhage | 55 | 3 | 0 | Y |
|  | 56 | 3 | 1 | Y |
| Tenesmus | 57 | 3 | 1 | Y |
|  | 58 | 2 | 1 | Y |
| Bowel Obstruction | 59 | 2 | 2 | N |
| Portal Hypertension | 60 | 3 | 3 | N |

Pain was evaluated via the Numeric Rating Scale (NRS, 0–10: 0=no pain, 1–3=mild, 4–6=moderate, 7–10=severe). Dyspnea, hemorrhage, intestinal obstruction, and ascites were graded per CTCAE v5.0. Tenesmus was graded 1–3 based on interference with sleep and daily living.
